# Health Benefits of Electrolyzed Hydrogen Water: Antioxidant and Anti-Inflammatory Effects in Living Organisms

**DOI:** 10.3390/antiox13030313

**Published:** 2024-03-02

**Authors:** Di Hu, Shigeru Kabayama, Yasuyoshi Watanabe, Yilong Cui

**Affiliations:** 1 Laboratory for Biofunction Dynamics Imaging, RIKEN Center for Biosystems Dynamics Research, Kobe 650-0047, Japan; hu-d@tachibana-u.ac.jp; 2 Department of Medical Technology and Sciences, Faculty of Health Sciences, Kyoto Tachibana University, Kyoto 607-8175, Japan; 3 Nihon Trim, Co., Ltd., Osaka 530-0001, Japan; kabayama@nihon-trim.co.jp; 4 Laboratory for Pathophysiological and Health Science, RIKEN Center for Biosystems Dynamics Research, Kobe 650-0047, Japan; yywata@riken.jp; 5 Department of Essential Healthcare Science, Graduate School of Science, Technology and Innovation, Kobe University, Kobe 650-0047, Japan; 6 Laboratory for Brain-Gut Homeostasis, Hyogo Medical University, Nishinomiya 663-8501, Hyogo, Japan

**Keywords:** molecular hydrogen, electrolyzed hydrogen water, antioxidant effect, anti-inflammatory effect, analgesic effect

## Abstract

Molecular hydrogen, the smallest and lightest molecule, serves as an intense reducing agent. Its distinct characteristics, including minimal size and neutral charge, enhance bioavailability and facilitate significant biological effects. Previously considered physiologically inert, hydrogen has gained recognition as a powerful therapeutic agent, known for its antioxidative and anti-inflammatory properties. Electrolyzed hydrogen water (EHW), enriched with molecular hydrogen, demonstrates remarkable antioxidative capabilities, indicating potential benefits for various diseases. Inflammation-induced reactive oxygen species (ROS) amplify inflammation, leading to secondary oxidative stress and creating a crosstalk between ROS and inflammatory responses. This crosstalk contributes to the pathogenesis and progression of chronic diseases. EHW interrupts this crosstalk, reducing inflammatory cytokines and oxidative stress across various disease models, suggesting therapeutic potential. EHW is also known for its anti-inflammatory effects, extending to pain management, as evidenced in models like sciatic nerve ligation and inflammatory pain. In an inflammatory bowel disease (IBD) model, EHW effectively alleviates abdominal pain, mitigating 2,4,6-trinitrobenzene sulfonic acid (TNBS)-induced inflammation and oxidative stress, offering insights for clinical applications. Additionally, hydrogen selectively targets harmful radicals, and EHW intake helps balance stress-induced hormonal dysregulation, potentially easing disorders associated with chronic stress.

## 1. Introduction

Molecular hydrogen is a distinctive physicochemical molecule. It is noteworthy that the unique characteristics of molecular hydrogen, including its small size, low mass, neutral charge, and nonpolar nature, grant it exceptional diffusivity among gases, allowing it to potentially penetrate cellular membranes [1,2,3]. Hydrogen has been shown to selectively scavenge reactive oxygen species and free radicals, such as hydroxyl radicals (^•^OH) and peroxynitrite (ONOO^−^) radicals. This property has led to its proposal as an effective treatment for various diseases, due to its antioxidant effects [4]. Hydrogen water, a convenient and practical alternative to daily hydrogen gas inhalation, particularly electrolyzed hydrogen water (EHW), provides health benefits by dissolving hydrogen at high concentrations. Recent research has uncovered its diverse biological impacts, primarily its antioxidant and anti-inflammatory effect on inflammatory pain [5] and inflammatory bowel disease [6]; its anti-apoptotic effect on gastric injury [7] and nuropathic pain [8]; and its anti-allergic effect on immediate-type allergy [9]. Over 100 clinical studies have demonstrated its therapeutic potential across a variety of diseases [10,11,12,13]. Although EHW and hydrogen-enriched water both exhibit antioxidative effects, EHW is more effective in scavenging reactive oxygen species (ROS), enhancing its health benefits. EHW interrupts the crosstalk between ROS and inflammatory responses, which significantly influences disease pathogenesis and contributes to chronic diseases, and reduces the initiation of further inflammation. It has shown anti-inflammatory and antioxidative effects in various disease models [6,14,15,16]. Notably, EHW has proven effective in different pain models, suggesting its potential in alleviating abdominal pain in conditions like inflammatory bowel disease (IBD) [6]. Additionally, EHW has demonstrated various protective benefits, such as mitigating oxidative stress, cognitive impairment, brain edema, and inflammation in diverse models [17,18,19].

This review will focus on the anti-inflammatory, analgesic, and anti-stress effects of electrolyzed hydrogen water. At this juncture, it is crucial to consider several aspects of electrolyzed hydrogen water in the context of this summary.

## 2. Properties of Hydrogen

Molecular hydrogen (H_2_) is a stable, neutral molecule consisting of two hydrogen atoms. It is colorless, odorless, non-toxic, and the smallest (lightest) substance in terms of density in its gas, liquid, or solid states [1]. Hydrogen’s electronegativity is higher than that of alkali and alkaline earth metals, but lower than that of halogens. Consequently, it functions as both an oxidizing and a reducing agent. This dual nature allows hydrogen to interact with both non-metallic and metallic elements. For example, in combustion reactions where hydrogen combines with oxygen atoms, it acts as a reducing agent to form water. Conversely, in reactions with sodium, it behaves as an oxidant, producing sodium hydride [2]. Studies utilizing tritium, a hydrogen radioisotope, have revealed that most hydrogen absorbed by mammalian cells is rapidly excreted from the body, and hydrogen is not oxidized in the organs [3]. A portion of hydrogen is converted to water, a process facilitated by microorganisms such as intestinal bacteria [20]. However, in aqueous solutions, hydrogen reacts with free radicals in the body.

The physicochemical properties of molecular hydrogen, such as its small size, low mass, neutral charge, and nonpolar nature, confer upon it the highest diffusivity among gases for penetrating cellular membranes, reaching even the mitochondria and nucleus [21,22]. These attributes enhance hydrogen’s effectiveness in its various biological interactions with living organisms. However, atmospheric hydrogen concentrations are less than 1 ppm (parts per million). While hydrogen gas can dissolve in water up to a concentration of 1.6 ppm, it does not alter the pH of the solution at room temperature and atmospheric pressure [23].

## 3. Antioxidant Effects of Hydrogen

Hydrogen was long considered a physiologically inert gas, believed to be non-reactive with living organisms. Hydrogen dose not react with biological compounds, including oxygen, at body temperature without a catalyst. However, certain enzymes known as hydrogenases in bacteria can utilize hydrogen as an energy source or as a byproduct of anaerobic metabolism [24]. In contrast, mammalian cells lack hydrogenase genes, leading to the assumption that hydrogen serves no function in mammals [20]. In 1975, Dole et al. reported that hydrogen gas significantly regressed skin tumors in animals, but this finding did not gain widespread attention [25]. A study in 2001 reported the anti-inflammatory effects of hyperbaric hydrogen in a mouse model of schistosomiasis-associated chronic liver inflammation [26], yet such studies were limited [27]. The perspective on hydrogen dramatically changed in 2007, when Ohsawa et al. demonstrated that hydrogen could scavenge highly reactive oxygen and nitrogen species, such as hydroxyl radicals (^•^OH) and peroxynitrite (ONOO^−^) in cells, thereby protecting them against oxidative stress [4]. This study overturned the conventional view of hydrogen and showed that inhaling hydrogen gas significantly reduced brain damage in rats following ischemia-reperfusion. Since then, there has been considerable interest in hydrogen’s antioxidant effects. Research, primarily using animal models, has investigated hydrogen’s therapeutic and preventive effects, resulting in the publication of over 1000 papers. These studies have demonstrated hydrogen’s efficacy in disease models where oxidative stress is a direct or indirect factor in nearly all organs. Additionally, hydrogen has been found to possess multiple functions, including anti-inflammatory [28], anti-apoptotic [29], and anti-allergic effects [9], and it stimulates energy metabolism [30,31]. Beyond model experiments, over 100 clinical trial papers have been published. Reports have also shown hydrogen’s effects on plants, expanding its application beyond medicine agriculture [32,33].

Daily inhalation of hydrogen gas is impractical for the continuous uptake of hydrogen in everyday life for disease prevention. In contrast, hydrogen water, where hydrogen is dissolved in high concentrations, offers a safe and practical alternative. It is portable, and hydrogen can be consumed simply by drinking. Hydrogen water is available in containers at drugstores and other outlets. There are also methods where magnesium sticks are added to bottled water to generate hydrogen, or water is electrolyzed using a generator or water purifier. Hydrogen escapes from glass and plastic containers quickly, but aluminum containers can retain hydrogen gas for extended periods. Although saturated hydrogen water has been more effective than diluted versions, even a 10-fold dilution of 80 μM hydrogen water has shown significant effects in the animals studies [30], indicating that lower hydrogen concentrations can be more effective than previously thought. Previous studies have reported no side effects from consuming high concentrations of hydrogen, as any excess is expelled via exhaled air [34,35,36]. Hydrogen levels of a few µM have been detected in the blood of rats administrated hydrogen intragastrically [23,37], and research is progressively revealing the effects of different consumption methods on hydrogen concentration changes in the body [38].

Using this approach, research on H_2_-dissolved water (hydrogen water) primarily began in the 1990s and revealed that there are numerous biologically beneficial effects of hydrogen water. These include antioxidative stress [10], suppression of lipid peroxidation [39], anti-inflammatory [40], neuroprotective [41], protection of DNA from oxidative damage [42,43], prevention of aspirin-induced gastric mucosal injury [7,44], anti-diabetic [30,45], and anti-cancer effects [46,47]. Among types of hydrogen water, the variety produced on the cathode side by the electrolysis of tap water using a water generator is known as electrolyzed hydrogen water (EHW). During electrolysis, hydroxyl ions and hydrogen gas are generated on the cathode side, resulting in the alkaline nature of EHW (pH 9~10). Conversely, on the anode side, hydrogen ions and oxygen gas are produced, creating acidic electrolytic water, or oxidized water (pH < 6.5). Compared to hydrogen water with an equivalent hydrogen concentration (0.9 ppm), EHW has been reported to possess approximately five times the ability to scavenge reactive oxygen species in HT1080 cells. Interestingly, even after the removal of dissolved hydrogen gas, approximately 60% of the scavenging activity remains in EHW [48].

Wang et al. [49] suggested that EHW contains certain amounts of platinum (Pt) clusters and/or Pt nanoparticles (over 2.2 ppb) released from Pt-based electrocatalysts (electrodes) during the electrolysis process. These free Pt clusters and/or Pt nanoparticles can convert hydrogen molecules (H_2_) into reductive hydrogen species (H·) through Pt/H2 catalytic interactions, thereby endowing EHW with distinctive reductive properties. As with hydrogen water, a wide range of effects has also been reported for EHW, including anti-inflammation in aspirin-induced gastric injury [50] and lipopolysaccharide (LPS)-induced inflammation models [51], inhibition of oxidative stress [17] and oxidative DNA damage [52], alleviation of abdominal pain in an IBD model [6], reduction of toxic acetaldehyde, and prevention of ethanol-induced cytotoxicity [53]. Additionally, EHW consumption in patients with type 2 diabetes has shown to improve insulin resistance [11], and in dialysis patients, it enhances quality of life by reducing fatigue [12] and lowering the risk of complications [13].

These reports suggest that EHW and hydrogen water share a common antioxidative effect in both basic and clinical research. The antioxidative capacity of EHW is attributed not only to its high levels of dissolved hydrogen, but also to the presence of a small amount of platinum nanoparticles originating from the platinum electrodes in the EHW production apparatus. Consequently, EHW may be a superior option compared to hydrogen-dissolved water for managing lifestyle-related diseases, cancer, aging, and other conditions that progress rapidly due to the production of a large amount of reactive oxygen species in living organisms (Table 1).

## 4. Anti-Inflammatory Effects of EHW

The crosstalk between ROS signaling and inflammatory responses has been extensively documented in various studies [55,56]. When the body encounters stimuli such as infection or trauma, the activation of inflammatory cells, notably neutrophils and macrophages, leads to increased respiratory bursts. This activity results in the production of primary ROS and reactive nitrogen species (RNS), which play a crucial role in defending against invading pathogens. Such processes trigger the release of primary cytokines by activating inflammatory transcription factors, particularly nuclear factor-kappa B (NF-κB). Concurrently, this can also stimulate the expression of nicotinamide adenine dinucleotide phosphate, reduced form (NADPH)-Oxygenase, which generates ROS from NADPH [55,57]. Proinflammatory cytokines further contribute to the accumulation of ROS in both phagocytic and nonphagocytic cells, leading to oxidative stress in various acute and chronic diseases [58].

The buildup of ROS plays a role in the proinflammatory differentiation of macrophages and the secretion of cytokines, whereas a reduction in ROS facilitates anti-inflammatory differentiation of macrophages and helps resolve inflammation [59]. Additionally, stimuli such as LPS, interferon-γ, and tumor necrosis factor (TNF)-α lead to the proinflammatory differentiation of macrophages and an increase in ROS production [59]. ROS are also known to promote the transcriptional activation of numerous inflammatory cytokines through the activation of signaling pathways like inducible nitric oxide synthase, cyclooxygenase (COX)-2, and the signal transducer and activator of transcription 3 [60,61]. Furthermore, primary cytokines stimulate inflammatory cells to produce secondary ROS, resulting in the additional release of secondary cytokines [58]. As intercellular ROS levels reach a toxic threshold, they can induce cell death through mechanisms such as necrosis or apoptosis, leading to the further recruitment of abundant inflammatory cells at the site of the lesions [62]. Therefore, inflammation and oxidative stress are concurrent and intricately linked processes in pathophysiology, with one often triggering the other.

Briefly, an increase in inflammatory cell-derived ROS exacerbates inflammation. ROS-dependent inflammation leads to secondary oxidative stress, creating a crosstalk. Such crosstalk between ROS and proinflammatory mediators can form a type of positive feedback loop, contributing to the pathogenesis and development of chronic diseases, such as type 2 diabetes [63], inflammatory bowel disease [64], and cancer [65]. These finding suggest that effective antioxidant interventions, which disrupt the crosstalk between ROS and proinflammatory mediators, could be beneficial in preventing the development of chronic diseases.

**Figure 1:**
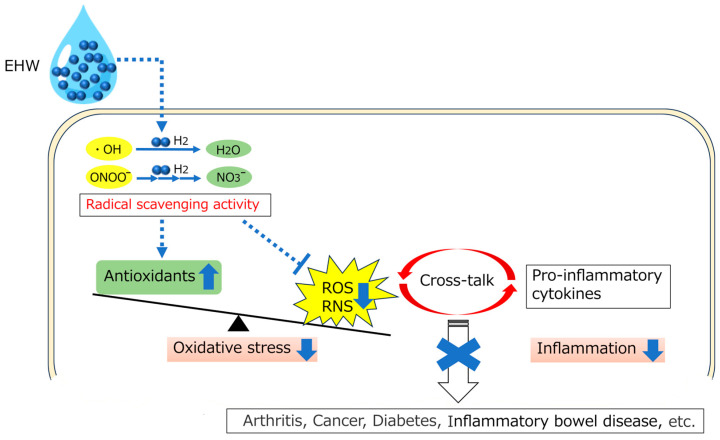
Schematic illustration of biological effects of EHW. EHW rectifies the imbalance of the redox state in the body by selectively scavenging ROS and free radicals, such as ^•^OH and ONOO^−^, and breaking the crosstalk between ROS and inflammation, thereby preventing the development of chronic diseases. EHW: Electrolyzed Hydrogen Water; ^•^OH: Hydroxyl radicals; ONOO^−^: Peroxynitrite; ROS: Reactive Oxygen Species; RNS: Reactive Nitrogen Species.

Several studies have demonstrated that the consumption of EHW suppresses the elevation of inflammatory cytokines and oxidative stress by disrupting this crosstalk [6,17]. For instance, EHW administration has been shown to suppress proinflammatory cytokines such as interleukin (IL)-1β, IL-6, and TNF-α, as well as excessive oxidative stress and calcium-binding protein (S100) A9 overexpression, which generates in neutrophils, monocytes, and dendritic cells during inflammation. This regulation suppressed the expression of cytokines via the NF-κB and activator protein-1 signaling pathways, a process closely associated with ROS generation, as seen in the 2,4,6-trinitrobenzene sulfonic acid (TNBS)-induced colitis model [6]. EHW also reduced TNF-α levels and restored glutathione in a dextran sulfate sodium-induced colitis model [14], inhibited proinflammatory cytokines such as IL-1β, IL-6, and COX-2 by suppressing NF-κB and activating the NF-E2-related factor 2 signaling pathway in an LPS-induced infection model [16], decreased levels of IL-1β and IL-33, and reduced mast cell infiltration in an atopic dermatitis model [15]. Additionally, it ameliorated increased serum amylase activity, neutrophil infiltration, lipid oxidation, and pancreatic tissue edema in an L-arginine-induced acute pancreatitis model [66], as well as in a collagen-induced arthritis model [67], among others. Furthermore, research has explored the consumption of EHW in patients with conditions like rheumatoid arthritis [10], atopic dermatitis [68], and end-stage renal disease [13,54] (Figure 1).

## 5. Analgesic Effects of EHW

With the anti-inflammatory properties of EHW gaining widespread recognition, its efficacy in diseases associated with inflammatory reactions, particularly in pain research, has been increasingly explored. Numerous reports have demonstrated the effectiveness of EHW in various pain models. For instance, in the partial sciatic nerve ligation model, EHW has been shown to suppress oxidative stress induced by ligation in the spinal cord and dorsal root ganglion [69]. In the chronic constriction injury (CCI) model, EHW helps to restore the level of antioxidant enzymes, like hemeoxygenase-1 (HO-1), modulating nerve-injury-induced neuropathy [8]. Furthermore, in the complete Freund’s adjuvant-induced inflammatory pain model, EHW has been found to upregulate the HO-1 and NADPH pathways, contributing to its analgesic activities [5].

Recently, Hu et al. reported that EHW alleviated abdominal pain associated with persistent colonic inflammation in a rat IBD model. This study demonstrated that EHW consumption suppressed TNBS-induced inflammatory responses and oxidative stress, thereby reducing abdominal pain in IBD [6]. The TNBS model is generated by colorectal administration of 2,4,6-trinitrobenzene sulfonic acid and ethanol to induce colitis, and is reported to have similarities to the pathogenesis of Crohn’s disease [70,71]. Using this model, the visceromotor response (VMR) to colorectal distension was recorded using electromyographic from the external oblique muscles, a method used to evaluate abdominal pain severity [72]. The VMR threshold significantly decreased following TNBS treatment, but gradually returned to pre-treatment levels within a few weeks. The consumption of EHW significantly inhibited the reduction in the VMR threshold, indicating that EHW consumption effectively relieves TNBS-induced abdominal pain. TNBS treatment induced localized ulceration in the intestinal tract, leading to the infiltration of inflammatory cells like neutrophils and monocytes into the submucosa and circular muscular layer. This infiltration is accompanied by a significant increase in myeloperoxidase secretion from neutrophils [73]. Additionally, the escalation of inflammatory cytokines such as IL-1β, TNF-α, IL-6, and chemokine monocyte chemoattractant protein 1 (MCP-1) from the intestinal tissue contributes to the development of an excessive cell-mediated immune response. This response further sensitizes afferent nerve terminals in the surrounding intestinal tract [74,75]. For example, IL-1β and TNF-α released from macrophages in inflamed tissue trigger the arachidonic acid cascade in fibroblasts and vascular endothelial cells to induce COX-2 upregulation, leading to the production of prostaglandin E2 (PGE2). PGE2 binds to the EP1/EP2 receptor expressed on C-fiber nociceptors within inflamed colon tissue to evoke visceral hypersensitivity [76,77,78]. The visceral pain threshold in response to colorectal distension (CRD), a mechanical stimulus to the colon that has been widely used as a reliable method for evaluating visceral sensitivity, was significantly decreased after TNBS treatment, indicating that the increased levels of inflammatory mediators in inflamed colon tissue induce hypersensitivity of peripheral nociceptors, and decrease the CRD-induced VMR threshold after TNBS treatment. Similarly, elevated expression levels of transient receptor potential vanilloid 1 (TRPV1) and other members of the TRP family, key nociceptive sensors instrumental in peripheral hypersensitivity, have been reported in inflamed colon tissue. These sensors show enhanced responsiveness to the action of inflammatory mediators via second messenger actions (e.g., PKA, PKC, and p38 MAPK) on primary sensory neurons in the dorsal root ganglion [79,80,81,82]. Intriguingly, blocking the signaling cascade via neutralizing antibodies or IL-1R antagonist attenuates hyperalgesia in the colitis model [83,84], underscoring the crucial role of inflammatory pathways in pain modulation. Moreover, chemokines such as MCP-1 bind to chemokine receptor CCR2 in peripheral neurons, leading to pain hypersensitivity via activation of TRPV-1, and directly excite primary nociceptive neurons within sacral dorsal root ganglia [85]. Conversely, CCR2 receptor antagonists mitigate visceral hypersensitivity by inhibiting the downstream of MCP-1/CCR2 signaling [86]. Consistent with these observations, the TNBS-induced decrease in the CRD-induced visceral pain threshold was significantly alleviated by consumption of EHW, suggesting its potential in reducing the production of inflammatory mediators in inflamed colon tissue, thereby preventing the development of peripheral nociceptor hypersensitivity, thus alleviating abdominal pain.

Furthermore, several reports demonstrated that ROS and their reactive products contribute to persistent pain, including inflammatory pain through various mechanisms [87,88]. ROS scavengers, such as phenyl-N-t-butyl nitrone (PBN) and 5,5-dimethylpyroline N-oxide (DMPO), have exhibited efficacy in alleviating pain responses [89]. For example, systemic administration of PBN has been shown to significantly inhibit TRPV-1 expression via TNF receptor type 1 (TNFR1) and prevent hyperalgesia [88]. Additionally, elevated ROS levels and their reactive products have been implicated in the upregulation of inflammatory mediator expression through the activation of the NF-κB signaling pathway [90,91]. Concurrently, the increased expression of inflammatory mediators can active NADPH-Oxygenase (NOX), facilitating ROS generation from NADPH during respiratory bursts in inflammatory cells within inflamed tissue [92]. This type of complicated crosstalk between ROS and inflammatory mediators forms a positive feedback loop that may contribute to the development of IBD [55,56,93]. A study based on a gastric injury model demonstrated the potent free-radical scavenging properties of EHW, effectively removing cytotoxic ROS and diffusing rapidly across membranes to protect stomach tissue from aspirin-induced inflammatory injury [50]. Similarly, Hu et al. found that EHW consumption significantly suppressed the increment of the diacron reactive oxygen metabolites (d-ROMs) and restored superoxide dismutase activity in inflamed colon tissue following TNBS treatment [6]. Overall, these findings indicate that EHW mitigates ROS overproduction and interrupts the crosstalk between ROS and inflammatory mediators, thereby attenuating the pathophysiology of IBD and alleviating associated abdominal pain.

These observations suggest that anti-inflammatory interventions could effectively alleviate abdominal pain in IBD. This hypothesis is supported by the fact that consumption of EHW reduces the production of inflammatory mediators in inflamed colon tissue. Consequently, EHW consumption prevents the development of peripheral nociceptor hypersensitivity and alleviates abdominal pain [82]. Although most of these observational data are not sufficiently detailed to elucidate the underlying mechanisms, they provide important insights into applying the novel effects of EHW in clinical research.

## 6. Anti-Stress Effects of EHW

Physical and psychological stress stimuli trigger the secretion of adrenal glucocorticoids and increase metabolism. While increased metabolism alone generates ROS, glucocorticoids have been shown to play both direct and indirect roles in modulating the onset of oxidative stress [94]. These hormones significantly impact the higher functions of the brain, particularly influencing stress response neuronal centers such as the hippocampus, amygdala, and hypothalamus [95]. Prolonged stress leads to elevated glucocorticoid levels and ROS production, causing oxidative damage in the hippocampus, and subsequent impairment of cognitive functions [96]. ROS has been found to inhibit the ligand-stimulated nuclear translocation of the glucocorticoid receptor, and the glucocorticoid suppression of the proopiomelanocortin gene promoter activity in corticotroph cells is attenuated [97]. This suggests that increased ROS production in an oxidative redox state weakens the glucocorticoid negative feedback system to the hypothalamic–pituitary–adrenal (HPA) axis. These findings indicate that excessive oxidative stress in the brain is a key factor in HPA axis dysfunction. Therefore, alleviating oxidative stress in the hypothalamus could prevent stress-induced HPA axis disorders and depressive behaviors [98,99].

Continuous stress challenges induced several stress responses, which were alleviated by the consumption of EHW [17,23,40,100]. Generally, acute stress challenges increase systemic levels of adrenocorticotropic hormone (ACTH), which decrease after a period due to negative feedback mechanisms. These mechanisms involve glucocorticoids activating the glucocorticoid receptor (GR) in the hypothalamus and pituitary, inhibiting ACTH biosynthesis [97,101]. However, increased oxidative stress can impair GR translocation to the nucleus, potentially disrupting glucocorticoid negative feedback and leading to prolonged ACTH elevation [101]. Additionally, IL-1β has been shown to activate the HPA axis and induce ACTH release [102,103,104]. Consistent with these findings, Hu et al. reported that ACTH levels significantly increased following continuous stress challenges, suggesting that increased oxidative stress and plasma IL-1β levels might disrupt the negative feedback, causing prolonged HPA axis activation [17]. The suppression of ACTH elevation by EHW during these challenges supports the hypothesis that EHW’s antioxidative effect could reactivate the HPA axis’s essential negative feedback function.

Kil et al. found that enhanced oxidative stress activated the phosphorylation of the p38 mitogen-activated protein kinase cascade, associated with downregulation of steroidogenic acute regulatory protein activity and steroid synthesis in the adrenal gland of mice [105]. Consequently, corticosterone levels decreased after continuous stress exposure, and EHW consumption restored these levels to normal. This suggests that oxidative stress-induced inhibition of steroidogenesis acts as a mechanism disrupting the glucocorticoid-related negative feedback, and EHW consumption might counteract the negative regulation of the p38 mitogen-activated protein kinase cascade. Hypocortisolism, often reported under chronic stress conditions, lands in various stress-related disorders such as myalgic encephalomyelitis/chronic fatigue syndrome, fibromyalgia, other somatoform disorders, rheumatoid arthritis, and asthma, which may be related to this mechanism [106]. In summary, EHW may regulate the neuroendocrine negative feedback loop through its antioxidative effects, thereby normalizing stress-related hormone secretion. This suggests that EHW could alleviate adverse responses induced by chronic stress by suppressing the cascade downstream of ACTH.

## 7. Other Biological Effects of EHW

Excessive stress overload can disrupt brain function balance and cause significant damage to both the brain and body when stress levels surpass a certain threshold. The small size of molecular hydrogen enables it to potentially penetrate the blood–brain barrier and directly affect the central nervous system. Hou et al. reported that hydrogen water mitigated oxidative stress injury and cognitive impairment in a fluid percussion injury model [19]. Another study revealed that intraperitoneal injection of hydrogen-rich saline also provided similar neuroprotective effects after traumatic brain injury (TBI) induced by controlled cortical impact (CCI), with these protective effects being dose-dependent [107]. Dohi et al. reported that hydrogen water alleviated brain edema, blood–brain barrier disruption, and neuroinflammation in a CCI-induced TBI model [41]. Furthermore, Tian’s group, using the same model, found that administration of hydrogen water reduced the mortality rates and improved the cognitive function [108].

It has been observed that peripheral stimulation with LPS induces inflammation in the brain and fatigue-like behavior, while the consumption of hydrogen water improves activity and suppresses brain inflammation [51]. Studies have also shown that hydrogen water can inhibit cognitive memory impairment caused by the lateral intracerebroventricular administration of β-amyloid [109]. Mice subjected to prolonged restraint stress exhibit cognitive memory deficits, which are ameliorated by the consumption of hydrogen water [23]. Sato et al. conducted studies on vitamin C-deficient senescence-accelerated mice, specifically through hypoxia/reoxygenation loading on brain slices. They found that superoxide levels in the brain decreased following the administration of hydrogen water [110]. It has also been reported that providing hydrogen water to aging-accelerated, senescence-accelerated mouse prone 8 mice mitigated cognitive memory impairment and degeneration of hippocampal neurons [18]. Furthermore, the intake of hydrogen water in these mice prevented cognitive memory impairment and hippocampal neuronal degeneration, coinciding with increased brain serotonin levels and enhanced serum antioxidant activity. Therefore, hydrogen water demonstrates a range of disease-suppressive effects in the central nervous system, effects that cannot be solely attributed to the reduction of reactive oxygen species/radicals by molecular hydrogen.

Additionally, it has been reported that the consumption of hydrogen water mitigates radiation damage to the mouse intestinal endothelium [111] and skin [112]. In a noteworthy study, Kawasaki et al. found that culturing pluripotent bone marrow stromal cells in the presence of hydrogen water inhibited cellular senescence without reducing in oxidative stress [113].

## 8. Conclusions and Perspectives

EHW is promoted for its potential health benefits, primarily due to its claimed antioxidant properties. However, as of now, the majority of studies on EHW, as detailed in this review, are based on animal models. Clinical research and detailed molecular studies exploring the mechanism of action of EHW remain sparse. While EHW is recognized for its diverse therapeutic effects, its practical application hinges on demonstrating efficacy surpassing existing therapies. It is crucial to ascertain the diseases for which molecular hydrogen is most effective, as well as the appropriate dosage and method of administration for optimal results. Without such clarity, clinical studies risk producing inconclusive outcomes. Additionally, a more comprehensive understanding of the mechanisms behind EHW’s effects is essential for its advancement in therapeutic applications.

## Figures and Tables

**Table 1:** Summary of the health benefits of EHW.

Benefits	Subject	Outline of Effects	References
Antioxidant Effects	IBD model rats	ROS↓, antioxidant defense↑	[6]
	Stress model rats	ROS↓, antioxidant defense↑	[17]
	In vitro	ROS↓	[42,43,46,47,48,52]
	Gastric injury model rats	Lipid peroxidation↓	[50]
	In vitro	Ethanol-induced cytotoxicity↓	[53]
	End-stage renal disease patients	Antioxidant defense↑	[54]
	Neuropathic pain model mouse	Antioxidant defense↑	[8]
	Inflammatory pain model mouse	Antioxidant defense↑	[5]
Anti-inflammatory Effects	IBD model rats	Inflammatory cell infiltration↓,	[6]
		Proinflammatory cytokines↓	
	Atopic dermatitis model mouse	Chemokine↓, cytokines↓	[15]
		Infiltration of mast cells↓	
	Stress model rats	Proinflammatory cytokines↓	[17]
	Gastric injury model rats	Proinflammatory cytokines↓	[7,50]
	Neuroinflammation model mouse/In vitro	Proinflammatory cytokines↓	[51]
		Anti-inflammatory cytokines↑	
		Microglial activation↓	
	End-stage renal disease patients	Inflammatory markers↓	[54]
	Inflammatory pain model mouse	Inflammatory mediator↓	[5]
Analgesic Effects	IBD model rats	Abdominal pain↓	[6]
	Neuropathic pain model mouse	Allodynia and hyperalgesia↓	[8]
	Inflammatory pain model mouse	Mechanical allodynia↓, Thermal hyperalgesia↓	[5]
Other Effects			
Anti-fatigue effect	Neuroinflammation model	Sickness behavior↓, recovery↑	[51]
Anti-apoptotic effect	Gastric injury model rats	Epithelial cell apoptosis↓	[7]
	In vitro	Neuronal cell apoptosis↓	[52]
	Neuropathic pain model mouse	Cellular death marker↓	[8]
Anti-diabetic effect	Type 2 diabetes patients	Lactate↓	[11]
Anti-tumor effect	In vitro	Tumor growth and invasion↓	[46]
		Tumor cell transformation↓	[47]

EHW: Electrolyzed Hydrogen Water; IBD: Inflammatory Bowel Disease; ROS: Reactive Oxygen Species.
